# Beneficial Effects on Brain Micro-Environment by Caloric Restriction in Alleviating Neurodegenerative Diseases and Brain Aging

**DOI:** 10.3389/fphys.2021.715443

**Published:** 2021-11-25

**Authors:** Li Zhang, Huachong Xu, Ning Ding, Xue Li, Xiaoyin Chen, Zhuangfei Chen

**Affiliations:** ^1^Key Laboratory of Central CNS Regeneration (Ministry of Education), Guangdong-Hong Kong-Macau Institute of CNS Regeneration, Jinan University, Guangzhou, China; ^2^Center for Brain Science and Brain-Inspired Intelligence, Guangdong-Hong Kong-Macao Greater Bay Area, Guangzhou, China; ^3^Bioland Laboratory, Guangzhou Regenerative Medicine and Health Guangdong Laboratory, Guangzhou, China; ^4^Neuroscience and Neurorehabilitation Institute, University of Health and Rehabilitation Sciences, Qingdao, China; ^5^School of Traditional Chinese Medicine, Jinan University, Guangzhou, China; ^6^Medical College, Kunming University of Science and Technology, Kunming, China; ^7^School of Sports Medicine and Health, Chengdu Sport University, Chengdu, China

**Keywords:** brain aging, extracellular microenvironment, neuroinflammation, cognitive functions, metabolic homeostasis

## Abstract

Aging and neurodegenerative diseases are frequently associated with the disruption of the extracellular microenvironment, which includes mesenchyme and body fluid components. Caloric restriction (CR) has been recognized as a lifestyle intervention that can improve long-term health. In addition to preventing metabolic disorders, CR has been shown to improve brain health owing to its enhancing effect on cognitive functions or retarding effect on the progression of neurodegenerative diseases. This article summarizes current findings regarding the neuroprotective effects of CR, which include the modulation of metabolism, autophagy, oxidative stress, and neuroinflammation. This review may offer future perspectives for brain aging interventions.

## Introduction

Aging is a cellular process that aims to eliminate cells with irreversible functional deficits, which prevents severe tissue damage, such as malignant tumors (Campisi and d’Adda di Fagagna, 2007). However, the accumulation of cellular aging may induce a series of pathological conditions, such as tissue degeneration, chronic inflammation, and neurodegenerative diseases. Specifically, aging is frequently associated with brain degeneration, which can be attributed to the morphological and functional impairments of neurons and glial cells. In brain tissue, current knowledge is primarily concerned with neuronal aging based on its limited regeneration capacity. However, the aging process also occurs in glial cells with the potency of *de novo* proliferation. Therefore, it is equally important to understand the senescence of glial cells during brain aging.

Typical features of aging cells include the disruption of normal gene expression, metabolic rhythm, and morphology, which develops into a proinflammatory phenotype called senescence-associated secretory phenotype (SASP; Sikora et al., 2021). Subsequently, the alteration of the secretory matrix may affect the extracellular environment, where homeostasis is critical for cell proliferation, differentiation, metabolism, and function. Under aging or disease conditions, the disruption of the cellular microenvironment induces pathological changes in cells. In mammalian brains, the microniche surrounding neural tissues consists of the extracellular matrix and the cerebrospinal fluid (CSF; Valiente et al., 2018), which are produced by the choroid plexus and circulate between the cerebral ventricles, pia mater, and the arachnoid space. The altered status of the microenvironment in one brain region can therefore be transmitted to distal regions by CSF circulation.

Caloric restriction (CR) is effective in extending the lifespan of numerous species, including mammals, and can prevent or delay the progression of various aging-related diseases (Bishop and Guarente, 2007; Halagappa et al., 2007). In rodents, brain aging and neurodegeneration are closely correlated with cellular energy metabolism, and CR treatment can rapidly elevate brain metabolite concentrations; moreover, these effects can persist for a long duration (Yanckello et al., 2019). For example, transgenic mice with obesity gene knock-in presented with more severe Alzheimer’s disease (AD)-like pathology and cognitive degeneration (Takeda et al., 2010). However, CR intervention can prevent the synthesis and deposition of amyloid-beta (Aβ) proteins via facilitating the proteolysis of Aβ (Wang et al., 2005). Similar benefits have been observed in acute cerebral injury, such as stroke or epilepsy (Ciobanu et al., 2017). Furthermore, recent research has demonstrated the potential role of the gut–brain axis in neurodegenerative diseases, such as AD, Parkinson’s disease (PD), and multiple sclerosis (MS; Fang, 2016; Cattaneo et al., 2017; Shahi et al., 2017). In particular, CR can affect the composition of gut microbes (Rangan et al., 2019), which prominently mediate peripheral energy homeostasis as well as central appetite signaling. Moreover, cytokines and metabolic byproducts from gut microbial populations can affect mental and cognitive behaviors via the vagal afferent nerve or neuroendocrine network (Zhou et al., 2019). In sum, CR can regulate the progression of neurodegeneration via both central and peripheral pathways.

In the following sections, we summarize current findings of CR in improving aging-related cellular microenvironments at the molecular, cellular, and tissue levels. This review aims to provide future perspectives for the application of CR in improving the brain microenvironment.

## Disruption of the Microenvironment by Brain Aging and Neurodegeneration

**FIGURE 1:**
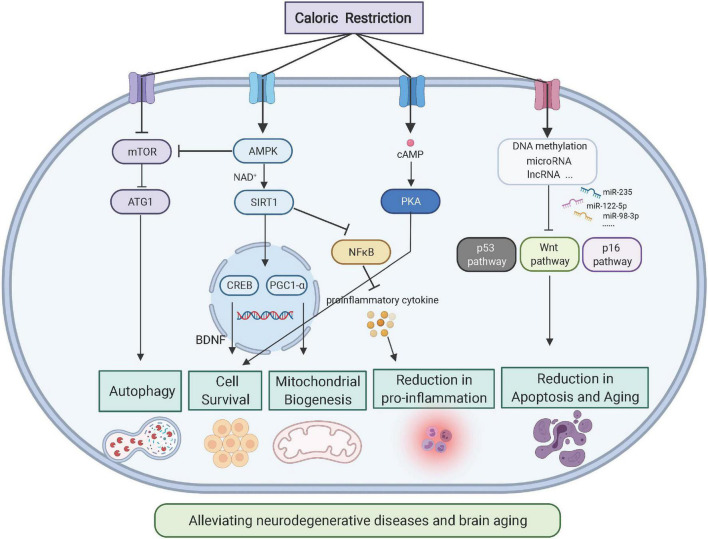
Relationship between aging/neurodegenerative diseases and the brain microenvironment (Created with BioRender.com). Multiple factors, such as neuroinflammation or permeability of the blood-brain barrier (BBB), may interact with the brain microenvironment to impact the progression of brain aging or neurodegenerative diseases. Such pathological changes can be attributed to the epigenetic mechanisms of metabolic waste. TBI, traumatic brain injury; MS, multiple sclerosis; AD, Alzheimer’s disease; PD, Parkinson’s disease; HD, Huntington’s disease; and ALS, amyotrophic lateral sclerosis.

Aging and neurodegenerative diseases frequently disrupt the homeostasis of the brain microenvironment, which further accelerates disease progression. The aging process involves multiple physiological aspects, which include stress adaptation, inflammation, metabolism, macromolecular damage, protein balance, epigenetic modification, stem cells, and tissue regeneration, all of which are associated with neurodegenerative diseases. Here, we focus on specific changes in the brain microenvironment under both aging and neurodegenerative disease conditions: inflammation, metabolism, permeability of the blood-brain barrier (BBB), and epigenetic modification (Figure 1).

### Inflammation and the Brain Microenvironment

Aging cells can synthesize and secrete various amounts of inflammatory modulators, proinflammatory cytokines, growth factors, chemokines, and proteinase, all of which contribute to the SASP (Birch and Gil, 2020). The primary components of the SASP include proinflammatory cytokines, such as interleukin (IL)-6, IL-8, and IL-1α, as well as immune-related chemokines that can bind with CXC receptors, which include CXCL-2, CXCL-3, CXCL5, and CCL2, which has high affinity with the CC receptor (Coppé et al., 2010; Calsolaro and Edison, 2016). Cells at the senescence stage can secrete SASP-induced cytokines into the extracellular environment, which triggers the immune cascade to remove themselves and forms a “cellular suicide” pathway.

During brain aging, elevated levels of cytokines contribute to chronic inflammation, which is primarily mediated by microglial cells. Inflammation can be induced by cytokines, such as IL-1β, IL-17, tumor necrosis factor-alpha (TNF-α), and IL-6 (Del Giudice and Gangestad, 2018). The IL-1 family facilitates the central inflammatory response given the wide distribution of its receptor (IL-1R) across brain tissues. Following ligand binding, the IL-1R activates downstream cascade pathways to induce nuclear factor kappaB (NF-κB), which elevates gene expression levels related to immune activation (Dinarello, 2019). IL-6 is a multipotent cytokine that can induce B cell differentiation or antibody production as well as T cell differentiation. In the brain, IL-6 activates microglial cells to promote neuroinflammation (Willis et al., 2020). As one of the most important immediate inflammatory mediators, TNF-α recruits neutrophils and lymphocytes and elicits the synthesis of other cytokines. In neurological diseases, TNF-α inhibits microglial autophagy via the Akt/mechanistic target of rapamycin (mTOR) pathway, which aggravates neuroinflammation (Jin et al., 2018).

As the sole innate immune cell type, microglia are widely distributed throughout the brain. Three major physiological functions are executed by microglial cells: sensing the extracellular microenvironment, coordinating homeostasis of neurons and glial cells, and defending against exogenous or endogenous attack (Hickman et al., 2018). Under pathological conditions, such as cerebral injury, neuroinflammation, and neurodegenerative diseases, microglial cells exhibit cellular proliferation and morphological changes and display unique phenotypes to exert neuroprotective effects. During the aging process, the microglial population displays specific transformation patterns toward a disease state, as demonstrated by a recent single-cell sequencing approach (Mrdjen et al., 2018).

Microglial cells mediate the neuroinflammatory status in the brain, which prominently affects the pathological process during brain aging and neurodegeneration. Specifically, microglia respond to adverse stimuli, such as protein aggregates, and exhibit cell toxicity to induce neuronal loss via specified pathways, such as the triggering receptor expressed on myeloid cells 2 and C-X3-C motif chemokine receptor 1 (Hickman et al., 2018). As the most widely studied mechanism, activated microglial cells can secrete various proinflammatory cytokines, such as TNF-α and IL-6, which results in cellular damage (Colonna and Butovsky, 2017). Moreover, microglia may propagate inflammatory processes due to cellular events, such as mitochondria fragmentation (Joshi et al., 2019). Under neurodegenerative conditions, the behavior of microglial populations is also affected by other immune cells, such as Th17 cells, which secrete IL-7 (Liu Z. et al., 2019). Furthermore, apart from neurodegeneration, the brain aging process is accompanied by dramatic changes in microglial populations and the neuroinflammatory environment. To support this, a transcriptomic study revealed a strong correlation between up-regulated microglial genes and the inflammatory process (Pan et al., 2020). Such transcriptomic modulation leads to a network shift of cellular pathways, which include the nuclear factor (NF)-kappa B, NLR family pyrin domain containing 3, and Caspase 1 pathways (Thawkar and Kaur, 2019), all of which contribute to neuronal loss. Taken together, microglial-mediated neuroinflammation is critical in reshaping the cellular microenvironment to participate in neurodegeneration.

### Metabolic Waste in the Microenvironment

Brain tissue consumes more than 20% of the oxygen and glucose within the entire body, despite only occupying approximately 2% of the total body mass, which illustrates its extremely high metabolic demand. Thus, this substantial amount of energy expenditure requires an efficient system for metabolic waste clearance. Without one, the deposition of byproducts disrupts the normal microenvironment and causes brain damage. In aging brain tissue, the imbalance in metabolism may cause oxidative stress and deposition of free radical species, both of which severely affect the extracellular microenvironment. For example, free radical species degrade the lipid bilayer of the membrane, resulting in the leakage of extracellular ions and consequently cell death, which produces further free radicals to aggravate the pathological condition (Cobley et al., 2018). During the aging process, the activity of antioxidants in the body gradually reduces, which results in higher sensitivity toward reactive oxygen species (ROS). Although specific neurodegenerative diseases possess unique protein factors, such as Aβ, α-synuclein, TAR DNA binding protein 43, and the huntingtin protein, these proteins present aggregates that disrupt normal neurological functions (Boland et al., 2018), which lead to ROS deposition. Thus, oxidative stress is recognized as one of the most important pathological factors across various neurodegenerative diseases.

The specific pathway by which protein metabolic byproducts are cleared from the extracellular environment of neural tissues is currently not fully understood. Traditional views neglect the existence of a lymphatic vessel system inside the brain and attribute waste degradation to cellular autophagy and the ubiquitination process, alongside the transport of specific biomolecules across the BBB. However, these pathways have relatively low efficiency that likely does not satisfy the high metabolic demand of the brain. Recently, *in vivo* imaging has consistently demonstrated the existence of a lymphatic system in the brain (Iliff et al., 2012, 2013). Specifically, brain astrocytes were shown to extensively express aquaportin-4 (AQP4), which helps the inflow of CSF into the brain to remove metabolic waste. The lymphatic vessel and arachnid network drain the CSF and form a lymphatic pathway for waste clearance (Rasmussen et al., 2018). Therefore, the functional interrogation of the brain’s lymphatic system may provide new insight into the disruption of the microenvironment during the aging process.

### Permeability of the Blood-Brain Barrier Affects the Brain Microenvironment

The brain was once considered an immune privilege zone because the BBB restricts the infiltration of circulating lymphocytes. However, recent studies have reported the entry of blood-derived immune cells into brain tissue. When BBB permeability is disrupted under neurological diseases or during aging, serum proinflammatory cytokines enter brain tissues to induce neuronal loss (Chinta et al., 2015). Such inflammation-induced tissue injury may further increase the permeability of the BBB. For example, astrocytes express junction protein claudin-5 to maintain the integrity of the BBB. However, during chronic inflammation, microglial cells engulf the end-feed of astrocytes to impair normal BBB function, which suggests a dual function of microglial cells during neuroinflammation (Haruwaka et al., 2019). Under stroke conditions, neuroinflammation can disrupt the BBB, whereby blood infiltrates the brain matter to induce vascular edema (Yang et al., 2019). Specifically, cerebrovascular edema caused by acute ischemic stroke and traumatic brain injury (TBI) further exacerbates BBB dysfunction by disrupting the balance between transporter and ion channels (Luo et al., 2020). In mouse models of transient middle cerebral artery occlusion, the AQP4 expression level in astrocytes was rapidly up-regulated following stroke and correlated with the degree of edema over time (Ribeiro Mde et al., 2006). On the other hand, AQP4 knockout in mice revealed intact BBB structure, which resulted in significantly reduced mortality, infarction, and cerebral edema after stroke as well as improvement in long-term neurobehavioral performance (Manley et al., 2000; Yao et al., 2015). Moreover, the sulfonylurea receptor 1 (SUR1)- transient receptor potential melastatin 4 (TRPM4) pathway is closely associated with cerebral edema formation, and conversely, SUR1-TRPM4 inhibitors reduce brain swelling and improve clinical outcomes following acute ischemic stroke or TBI (Luo et al., 2020). In addition, recent research has demonstrated that SUR1-TRPM4 and AQP4 form a novel heterooligomer that amplifies ion/water osmotic coupling and drives astrocyte swelling (Stokum et al., 2018), which coordinate to affect brain edema.

Moreover, metabolic waste in the brain microenvironment is closely associated with the neurodegeneration process. Aging leads to the loss of protein homeostasis, and the interaction with extracellular vesicles is critical during the neurodegeneration process (Guix, 2020). The dysfunction of the autophagic or lysosomal complex deprives the normal potency of the timely removal of metabolic waste, which can result in the development of neurodegenerative diseases (Malik et al., 2019). From the perspective of the glymphatic system, dysregulated metabolic waste clearance may also disrupt the homeostasis of the brain microenvironment (Kylkilahti et al., 2021). In particular, metabolic disorders of brain tissues may also induce neuroinflammation and dysfunction of the BBB via multiple signaling pathways (Van Dyken and Lacoste, 2018), thus, regulating the homeostasis of the microenvironment. In sum, the BBB is critical for maintaining the homeostasis of the brain microenvironment, which is also affected by inflammation and metabolism. Future studies of BBB functional integrity during aging are critical to improving our understanding of the brain aging process.

### Epigenetics and the Brain Microenvironment

Epigenetics refers to the modulation of gene expression and body phenotype without the alteration of genomic information and includes DNA methylation, histone modification, non-coding RNA regulation, and chromatin remodeling. Changes in DNA methylation are the hallmark of aging during the life span (Xiao et al., 2016). Researchers have shown that aging-associated DNA hypermethylation occurs preferentially at CpG islands and bivalent chromatin domain promoters, which suggests that methylation at specific gene sites is a biomarker of aging (Tra et al., 2002; Rakyan et al., 2010). A clinical study revealed that various neurodegenerative diseases, such as AD, dementia, and PD, exhibit similar patterns of abnormal DNA methylation (Sanchez-Mut et al., 2016). In AD patients, Chouliaras et al. (2012) observed an overall decrease in methylation levels in the hippocampus. Moreover, histone methylation modifiers alter chromosome structure to regulate gene expression, which impacts the progress of age-related diseases by regulating the expression of key age-related genes, such as P16 and telomere length (McCauley and Dang, 2014).

Furthermore, non-coding RNA, including microRNA and long non-coding RNA (lncRNA), affects aging-related gene expression. Clinical studies have revealed altered expression levels of multiple microRNAs in the blood of aging people, and the biological functions of these differentially regulated microRNAs include cell growth, development, and aging by regulating the expression of the telomerase, p53, and p16 signaling pathways (Pourrajab et al., 2015). Therefore, the role of lncRNA in aging and aging-related diseases should not be underestimated. For example, lncRNA-p21, which is a key target of the p53 signaling pathway, plays a role in regulating age-related diseases (Yang et al., 2016).

## Mechanisms of Caloric Restriction in Improving the Cellular Microenvironment

We briefly summarized the effect of various factors on the brain microenvironment during aging and neurodegeneration. Based on this, different drugs and non-drug protocols may help restore homeostasis of the brain microenvironment to recover neural functions. As an extensively studied lifestyle strategy, CR has been interrogated for its potential role in alleviating aging-related cognitive deficits. For example, CR in aging mice protects against neural progenitor cell loss, likely via alleviating chronic inflammation (Apple et al., 2019). In the following sections, we discuss the potential mechanisms of CR for improving extracellular homeostasis to recover aging-related deficits at the molecular, cellular, and tissue levels.

### Molecular Level

#### Inhibition of the Energy Metabolic Sensory Mechanistic Target of Rapamycin- Adenosine Monophosphate-Activated Protein Kinase Pathway

The mTOR and insulin receptor pathways are critical for sensing cellular energy metabolism and coordinate anabolism, which leads to the potentiation of protein or lipid synthesis, ribosome neogenesis, mitochondrial metabolism, cell growth, and mitosis (Ben-Sahra and Manning, 2017). Under excess nutritional status, tissue mTOR is hyperactivated, which results in a change in protein catabolism and an elevated production of mitochondrial ROS; simultaneously, autophagy is inhibited, which results in an inflammatory response (Kapahi et al., 2010). Therefore, a persistently activated mTOR pathway accelerates the aging process, and the inhibition of mTOR signaling mimics the effect of CR in potentiating the immune response and extends the lifespan (Harrison et al., 2009; Selman et al., 2009). Adenosine monophosphate-activated protein kinase (AMPK) is a signaling protein for sensing low-energy status, and it can inhibit the mTOR cascade reaction by phosphorylating the tuberous sclerosis complex 2 (TSC2) complex (Inoki et al., 2003). As the “hungry sensor” of cells, the AMPK pathway responds to low-energy levels to antagonize the aging process (Mair et al., 2011; Martin-Montalvo et al., 2013; Hardie, 2015) and is related to CR-induced neuroprotective effects (Ma et al., 2018). Thus, the AMPK and mTOR pathways antagonize each other to maintain the metabolic homeostasis of tissues.

Both mTOR and AMPK pathways play important roles in the pathogenesis of aging-related neurodegenerative diseases and are responsive to CR. For example, the deposition of Aβ proteins has been shown to activate neuronal mTOR, which facilitates *de novo* protein synthesis and inhibits autophagy, accelerating the aggregation of Aβ and Tau proteins. Therefore, as a specific mTOR inhibitor, rapamycin can improve cognitive functions, as demonstrated in AD animal models (Caccamo et al., 2010). Similarly, the inhibition of mTOR can induce cell autophagy to relieve disease symptoms (Ravikumar et al., 2004), and food restriction and exercise training have been shown to improve syndromes (Mattson, 2012). A further mechanistic link was provided by the induction of neuronal AMPK phosphorylation by CR via the upregulation of fibroblast growth factor 21 to suppress mTOR activity and relieve the hyperphosphorylation of tau proteins (Rühlmann et al., 2016). In examinations of Aβ, although no study has directly investigated the relationship between CR and Aβ deposition, the known effect of AMPK activation in relieving Aβ aggregates (Chen et al., 2021) and its potentiation under CR (Cantó and Auwerx, 2011) indicates that Aβ pathology may be relieved by reducing calorie intake. Thus, the regulation of cellular homeostasis by mTOR and AMPK prevents the body from metabolic imbalance (González et al., 2020). Moreover, both AMPK and mTOR play important roles in the hypothalamic regulation of body metabolism (Martínez de Morentin et al., 2014). However, further studies are needed to understand how the coordination between these two molecular pathways influences energy metabolism and the brain aging process.

#### Sirtuin and Oxidative Stress in Neuroprotection Against Aging

Sirtuin is an evolutionally conserved family of deacetylase that plays specific roles in extending the lifespan (Bishop and Guarente, 2007). Allosteric regulation of nicotinamide adenine dinucleotide (NAD) activates Sirtuin, which further affects histones and transcription factors to regulate gene expression, and CR can also activate this deacetylase to relieve oxidative stress (Qiu X. et al., 2010). Moreover, Sirtuin works synergistically with other nutrition-sensitive proteins that are activated by CR, such as AMPK (Cantó et al., 2009; Price et al., 2012) and peroxisome proliferator-activated receptor-gamma coactivator 1 alpha (PGC-1α; Cantó and Auwerx, 2009), to exert neuroprotective effects. Moreover, CR has been reported to relieve post-surgical memory deficits in aging mice by activating deacetylase to relieve endoplasmic reticulum stress in the hippocampus (Yao et al., 2019). Furthermore, the effect of Sirtuin on the cellular microenvironment has been demonstrated by previous studies that showed that the activation of cellular autophagy (Lee et al., 2008) and the inhibition of neuroinflammation dependent on NF-κB (Yeung et al., 2004) relieves neurogenerative diseases.

#### Feedback of the cAMP-Responsive Element-Binding–Sirtuin1 Axis by Caloric Restriction

Different environmental and internal stimuli, such as nutrition, hormones, and growth factors, can activate the cAMP-responsive element-binding (CREB) protein family, which profoundly affects the expression of brain-derived neurotrophic factor (BDNF) to mediate the survival, growth, differentiation, and plasticity of neurons (Lonze and Ginty, 2002). CREB also modulates the transcriptional activation of tyrosine receptor kinase B, which is the major receptor for BDNF (Tabuchi et al., 2002). CR potentiates CREB expression and activation via the AMPK cascade pathways; thus, CREB is a hub for nutritional sensors and neuronal growth. It has been found that a deficiency in the CREB pathway in mature neurons reduces the response of mice toward 30% CR, which is in stark contrast to wild-type mice, in which cognitive functions and electrophysiological parameters were enhanced after CR. However, CREB can directly activate Sirtuin1 in the mouse hippocampus (Fusco et al., 2012). In neurodegenerative diseases, Sirtuin1 can potentiate CREB-target of rapamycin complex 1 transcriptional activity to improve disease symptoms (Jeong et al., 2011). Therefore, the CREB–Sirtuin1 axis is critical for multi-layered brain responses under CR; however, upstream hormonal factors that induce the CREB cascade require further investigation.

#### Effects of Caloric Restriction on Neurotrophic Factors

Neurotrophic factors (NTFs) are a group of naturally occurring proteins that regulate the development, maturation, and functional integration of the nervous system. During CR, the expression level of NTFs, such as BDNF, neurotrophic factor 3 (NT3), glial cell line-derived neurotrophic factor (GDNF), and fibroblast growth factor 2 (FGF2), are increased in the brain, which is likely a compensatory mechanism against physiological stress (Qiu G. et al., 2010).

Numerous studies have shown that BDNF plays a critical role in mediating synaptic plasticity, neurogenesis, and neuronal stress resistance, especially during learning and memory. For example, the hippocampal BDNF level was positively correlated with the performance of a spatial learning task (Hall et al., 2000), whereas BDNF knockout mice showed impaired memory formation (Mizuno et al., 2000). In previous experiments, CR/intermittent fasting (IF) increased BDNF expression across different brain regions in rodents, including the hippocampus and cortex (Duan et al., 2001). In addition, Type 2 diabetes mellitus (T2DM) is known to be associated with an increased risk of dementia, and 12-week fasting in T2DM rats improves behavior and brain function by increasing the levels of NT3 and BDNF. NT3 is involved in the synaptic release of numerous neurotransmitters in both peripheral and central nervous systems and affects tissue development (Tyler et al., 2002). Similar to BDNF, GDNF has been shown to exert neuroprotective effects in primate PD models and is currently being used in clinical trials in PD patients (Gill et al., 2003). CR significantly increases the concentration of GDNF by almost three times that in the control group and leads to activated neuroprotective signaling pathways of DA neurons (Maswood et al., 2004). Both *in vivo* and *in vitro* studies have shown that GDNF and BDNF protect neurons from excitatory toxicity and metabolic and oxidative damage (Maswood et al., 2004). Moreover, other NTFs are likely related to brain aging, such as FGF2, which has been suggested to be involved in modulating synaptic plasticity during food deprivation, although its specific mechanisms remain unclear (Graham and Richardson, 2011; Mattson et al., 2018). Insulin-like growth factor 1 (IGF-1) is a phylogenetically ancient neurotrophic hormone that plays a crucial role in central nervous system development and maturation. Fasting mimicking diet (FMD) increases the expression of IGF-1 and its receptor in the dentate gyrus (DG) of the hippocampus and subsequently potentiates adult neurogenesis (Brandhorst et al., 2015). Taken together, the elevated NTFs induced by CR are essential for the effects of CR on brain aging and nerve regeneration.

#### Epigenetic Regulation of Caloric Restriction

Recent epigenetics research has revealed that CR affects the progression of aging and related diseases by regulating gene expression through epigenetic modifications (Molina-Serrano and Kirmizis, 2017). As an environmental stimulus, CR affects DNA methylation levels to alter chromosome structure, which modulates the expression of genes related to aging and neurodegenerative diseases (Hahn et al., 2017). With CR in aging mice, DNA methyltransferases (DNMT) 1 activity increases to relieve the hypomethylation of senescent DNA, whereas the DNMT3a expression level in the hippocampus changes to protect brain function (Chouliaras et al., 2012). Sirtuin 1 is a member of the NAD^+^–dependent histone deacetylase family. Numerous experiments have demonstrated the role of SIRT1 in mediating the anti-aging effect of CR, such as deacetylation effect of histones at H4K16 and H3K9 sites, to maintain gene silencing for resisting environmental stress and aging (Gong et al., 2015).

In addition to gene transcripts, non-coding RNAs, such as microRNAs and lncRNAs, are critical for maintaining homeostasis of the body as well as for the development of neurodegenerative diseases. CR increases the expression of microRNA necessary for mitochondrial protein translation (Zhang et al., 2019). CR markedly increases global and mitochondrial microRNA levels; moreover, microRNA-122 has been found to specifically activate mitochondrial translation (Zhang et al., 2019). In a second study, microRNA-235 was induced by CR to mediate body longevity via the Wnt signaling pathway (Xu et al., 2019). In brain tissues of CR-treated mice, the downregulation of three microRNAs, 34a, 30e, and 181a, helped maintain neuronal survival (Khanna et al., 2011). Recent studies (Khanna et al., 2011; Wood et al., 2015; Makwana et al., 2017; Xu et al., 2019; Zhang et al., 2019; Shastri et al., 2020; Yamada et al., 2020) have demonstrated that CR/dietary restriction (DR) regulates the expression of aging-related microRNA, as shown in Table 1. In examinations of lncRNA, CR helped protect cardiomyocytes by mediating lncRNA metastasis associate lung adenocarcinoma transcript 1 (MALAT1) and GAS5 (Rajagopalan et al., 2020). In addition, in a Drosophila model, lncRNAs mediated the aging pathway during CR treatment (Yang et al., 2016). In sum, non-coding RNAs participate in the process of cell growth, development, and aging primarily by affecting the p53 and P16 signaling pathways.

**TABLE 1:** CR/DR regulates the expression of aging-related microRNA.

**Model species**	**Diet scheme**	**miRNAs status**	**References**
Caenorhabditis elegans	DR	**↑**miR-235, miR-92b	Xu et al., 2019
C57BL/6J mouse	CR	**↑**miR-122-5p, miR-148a-3p, miR-192-5p, miR-101b-3p, miR-22-3p, miR-29a-3p, miR-let-7a-5p, miR-let-7c-5p, miR-126a-5p	Zhang et al., 2019
C57BL/6J mouse (brain)	CR	**↓**miR-181a-1*, miR-30e, miR-34a	Khanna et al., 2011
Male BN rats	CR	**↑**miR-98-3p	Wood et al., 2015
C57BL/6J mouse	CR	**↑**miR-16-5p, miR-196b-5p, miR-218-5p	Yamada et al., 2020
C57BL/6J mouse	CR	**↑**miR-29b, miR-29c, miR-30a, miR-30b	Shastri et al., 2020
C57BL/6J mouse	CR	**↑**miR-125a-5p, miR-145b, miR-466m-5p, miR-669m-5p, miR-6240, miR-466c-5p, miR-6931-5p, miR-455-3p, miR-139-5p, miR-466d-3p, **↓**miR-5130, miR-669h-3p, miR-6239, miR-132-3p, miR-6970-3p, miR-7056-5p, miR-212-3p	Makwana et al., 2017

### Cellular Level

#### Mitochondrial Biogenesis and Neuroprotection

When the body faces increased demands of oxidation or damaged mitochondria, mitochondrial biogenesis (MB) is induced via the PGC-1α pathway (López-Lluch et al., 2008). Two CR-related cellular metabolic sensors are AMPK and Sirtuin1, which modulate PGC-1α activity by phosphorylation and deacetylation, respectively. Mouse models have shown that PGC-1α inhibition relieves brain oxidative injury and neurodegeneration (St-Pierre et al., 2006). For example, the mutant huntingtin protein inhibits the transcription of PGC-1α to induce mitochondrial damage, which leads to oxidative stress and metabolic failure, whereas overexpression of PGC-1α prevents such cellular damage (Cui et al., 2006). CR can potentiate MB via the upregulation of nitric oxide synthase (NOS) to enhance cellular respiration to improving neuronal survival during brain aging (Nisoli et al., 2005). Moreover, MB participates in hippocampal synaptogenesis and plasticity (Wrann et al., 2013) and thus, exerts neuroprotective effects at the tissue level.

#### Cellular Autophagy Regulated by Caloric Restriction

**FIGURE 2:**
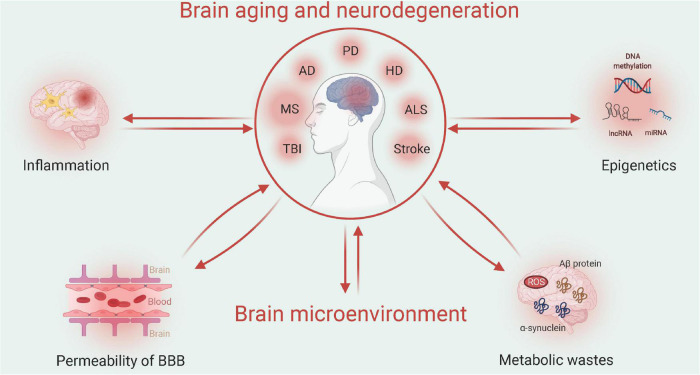
Molecular pathways of CR on neuroprotection (Created with BioRender.com). As the energy sensor, AMPK-mTOR pathways respond to CR by reacting to the metabolic status of cells. As the intracellular pathway, nicotinamide adenine dinucleotide-mediated sirtuin affects gene expression to generate neurotrophic factors such as BDNF. Downstream of Sirtuin 1, PGC-1α modulates mitochondrial biogenesis and function, which affects cellular metabolism.

Autophagy is a widely studied catabolic process in which damaged organelles or large biomolecules are encapsulated by autophagosomes, which are subsequently fused with lysosomes to digest cell debris (Mizushima and Komatsu, 2011). The dual roles of autophagy of cellular debris degradation and byproduct recycling provide substrates for the biosynthesis of large molecules as well as energy resources under conditions of nutritional deficiency (Mizushima and Komatsu, 2011). As a mechanism that counteracts energy insufficiency, autophagy restores cellular activity by clearing damaged mitochondria, abnormally folded proteins, and deposited lipids (Tschopp, 2011). For example, in an AD model, CR facilitated autophagy and activated glial cells to reduce neuronal Aβ loads (Gregosa et al., 2019). Moreover, recent studies have revealed that CR activates cellular autophagy in hepatocytes, adipocytes, skeletal muscle, and hypothalamic tissues, which indicates a close relationship between autophagy and CR (Martinez-Lopez et al., 2017). One study also showed that short-term fasting leads to a dramatic upregulation in neuronal autophagy (Alirezaei et al., 2010). A summary of the major molecular pathways of CR is provided in Figure 2.

#### Gut Microbes During Caloric Restriction-Induced Improvements in Cognitive Deficits

The brain–gut axis is a complex bidirectional communication system that includes neuroimmune, neuroendocrine, and neural pathways (Osadchiy et al., 2019). The communication between gut microbes and the central nervous system affects mood, behavior, and cognitive functions as well as the processes of aging and neurodegenerative diseases (Zarrinpar et al., 2014). Dietary factors, such as food types and dietary patterns, not only directly affect the central nervous system by influencing nerve cell membranes, neurotransmitters, and cerebrovascular functions but also interact with the central nervous system through the intestinal microbiome. Therefore, interventions of dietary style can significantly alter the composition and function of gut microbes (Attaye et al., 2020).

**FIGURE 3:**
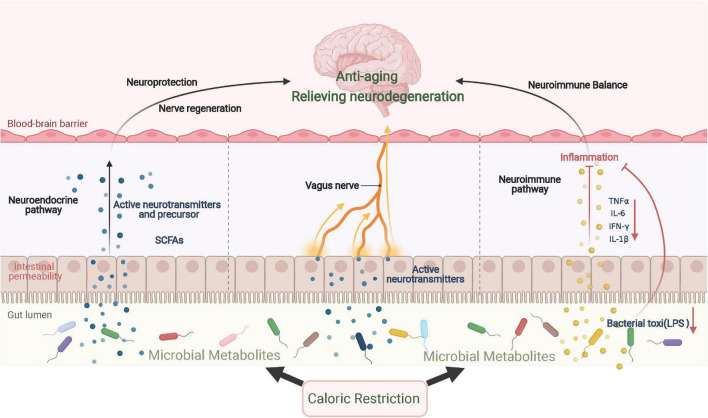
CR improves aging and neurodegenerative diseases via the gut–brain axis. CR significantly changes the composition and metabolism of gut microbes, which results in the production of various neurotransmitters and their precursors, inducing a neuroprotection effect. When inflammatory cytokine levels are repressed locally, neuroinflammation is further relieved. In addition to immune and endocrine regulation, gut microbes send signals directly to the central region via vagal afferent nerves. SCFAs, short-chain fatty acids.

Caloric restriction can alleviate neuroinflammation and neurodegeneration through intestinal flora and its metabolites, which then relieve the symptoms of aging and neurodegenerative diseases. On one hand, dietary patterns may alter the composition and metabolites of the gut microbiome, some of which activate immune cells and microglia in the brain to affect neuroinflammation (Zhang et al., 2020). Furthermore, CR can decrease the biosynthesis of lipid A, a critical component of lipopolysaccharides, and its downregulation further facilitates the infiltration of eosinophils that originate from adipose tissues and the polarization of anti-inflammatory macrophages (Fabbiano et al., 2018). In PD mice, FMD was shown to lower the number of glial cells to reduce the release of TNF-α and IL-1β by altering the intestinal flora and its metabolites (Zhou et al., 2019). On the other hand, CR can affect the metabolism of intestinal microorganisms, which alters the generation of neurotransmitters, neurotransmitter precursors, and other metabolites, such as serotonin, tryptophan, and short-chain fatty acids (SCFAs). Intestinal microbial metabolites have been widely recognized to affect emotional behavior, neurogenesis, and the integrity of the BBB (Luczynski et al., 2016). For example, SCFAs produced by the intestinal microbiota can cross the BBB, and butyrate has been found to exert neuroprotective effects by relieving neuroinflammation and increasing BDNF (Zhou et al., 2019). In addition, the intestinal and vagus nerves are important signal communication pathways in the gut–brain axis and warrant further study; the effect of CR remains unexplored. The possible mechanism of CR in alleviating aging and neurodegenerative diseases through the gut–brain axis is summarized in Figure 3.

### Tissue Level

#### Inhibition of Focal Inflammation by Caloric Restriction

Chronic inflammation is the common pathological feature of various metabolic disorders (Hotamisligil and Erbay, 2008). During the aging process, persistent systemic inflammation aggravates tissue degeneration (Franceschi and Campisi, 2014). Moreover, chronic inflammation induces metabolic disorders, such as insulin resistance, which leads to the disruption of neuronal functions and the acceleration of neurodegeneration (Heneka et al., 2013). In addition, neuroinflammation damages hypothalamic nuclei, which are the centers responsible for the homeostasis of energy and blood glucose; thus, causing obesity and diabetes (Jordan et al., 2010). The anti-inflammatory effects of CR have been demonstrated in both animal models and human patients (Mercken et al., 2013; Park et al., 2019). For example, CR and intermittent feeding relieve neuroinflammation in the rodent hypothalamus and hippocampus (Radler et al., 2014; Vasconcelos et al., 2014). CR also decreases serum and brain concentrations of proinflammatory cytokines via the suppression of NF-kB activity and elevates BDNF expression (Vasconcelos et al., 2014), which offers protective effects during acute brain damage (Manzanero et al., 2011) and brain aging.

#### Blood-Brain Barrier and Caloric Restriction

In addition to the suppression of neuroinflammation discussed above, CR also helps to maintain normal BBB permeability and function primarily by improving cerebral vascular homeostasis and the gut–brain axis. Recent studies have revealed a beneficial effect of CR on brain vascular health. Low caloric diets help to maintain blood vessel homeostasis, which includes the reduction of oxidative stress, enhancement of nitric oxide bioactivity, and the suppression of vascular inflammation. Studies on the molecular mechanisms showed that critical cytokines and pathways, such as AMPK, mTOR, and endothelial nitric oxide synthase (eNOS) pathways, all participated in the maintenance of vascular homeostasis (Liu et al., 2014). CR attenuates neuronal loss, induction of heme oxygenase-1, and BBB breakdown induced by impaired oxidative metabolism (Calingasan and Gibson, 2000). Furthermore, moderate CR has neuroprotective effects: it reduces p53-mediated neurovascular damage and microglia activation after hypoxic ischemia to maintain normal permeability of the BBB (Tu et al., 2012). However, the protein and ion channels mediated by CR responsible for the protection of BBB permeability require further study.

Both long- and short-term CR potentiates eNOS expression in endothelial vascular cells (Minamiyama et al., 2007). Such information from peripheral vessels indicates the beneficial effects of CR on cerebral vessels. In a high-fat diet mouse model, CR treatment significantly relieved BBB leakage (Kim et al., 2016). Another aging mouse model showed that CR significantly preserved cerebral blood flow at 20 months of age when cerebrovascular function should be compromised under normal feeding conditions (Parikh et al., 2016). In addition, intestinal flora promotes the integrity and functional stability of the BBB, and CR has been shown to have positive effects on intestinal flora. Specifically, CR promotes the expression of tight junction proteins in brain endothelial cells by changing SCFA production in the intestinal flora and thus maintaining the integrity of the BBB (Logsdon et al., 2018). These data illustrate the potentially important role of CR in maintaining the integrity of the BBB and cerebrovascular functions. Overall, the integrity and normal function of the BBB increase the stability of the brain microenvironment, which alleviates the symptoms of aging and neurodegenerative diseases.

#### Caloric Restriction-Mediated Enhancement of Neural Plasticity

Synaptic plasticity is critical for learning and memory functions (Mattson, 2012), which are dependent on synaptic density, morphology, and function. In rodents, brain aging is accompanied by weakened synaptic plasticity, as reflected by impaired long-term potentiation in the hippocampus, down-expression of synaptic proteins, and impaired BDNF expression or receptors. CR can prevent these aging-related adverse effects and improve cognitive deficits (Fontán-Lozano et al., 2008). Specifically, in mice, CR enhances cognitive functions by increasing the spine density of the hippocampal DG region (Wahl et al., 2018). Because synaptic plasticity is highly dependent on the mitochondrial activity at axonal terminals (Levy et al., 2003), CR can potentiate synaptic plasticity by enhancing NOS-dependent MB (Nisoli et al., 2005). Moreover, CR can also benefit synaptic plasticity by activating anti-inflammatory pathways under brain aging or acute cerebral injury conditions (Pani, 2015).

In addition to the enhancement of synaptic plasticity, CR may also facilitate adult neurogenesis, which occurs in specific brain regions including the subventricular zone (SVZ) and hippocampal DG nuclei. The constitutive proliferation and differentiation of neural progenitor cells generate both neurons and glial cells, which actively replace damaged cells and participate in the homeostatic regulation of the brain microenvironment and thus, play specific roles in learning and motor regulation (Zhang et al., 2008). It has also been shown that CR increases the expression of BDNF in the hippocampus (Kaptan et al., 2015), which elevates the number of proliferating neural progenitor cells and facilitates their differentiation toward mature neurons and glial cells (Park and Lee, 2011).

#### Effects of Caloric Restriction on Neural Autoimmunity

Neural autoimmunity refers to the autoimmune response that specifically targets the nervous system, leading to neurodegenerative diseases, such as MS and AD (Singh, 1997). During these responses, cellular immunity plays a key role in the development of autoimmunity and inflammation. For example, MS is characterized by T cell-mediated demyelination and neurodegeneration in the central nervous system (Friese and Fugger, 2005; Sospedra and Martin, 2005). In experimental autoimmune encephalomyelitis (EAE) animal models, activated myelin-specific TH1 and TH17 cells were shown to cross the BBB and migrate to the central nervous system, where they were subsequently activated by local antigen-presenting cells to promote inflammation (Dhib-Jalbut, 2007; Goverman, 2009). Recent studies have found that CR can reverse immunosuppression or immune aging associated with chemotherapy or hematopoietic stem cell-based regeneration (Cheng et al., 2014; Brandhorst et al., 2015). Similarly, animal experiments have indicated that FMD reduces the number of dendritic cells and increases the relative number of naive T cells, which reduces the infiltration of immune cells into the spinal cord (Choi et al., 2016). Moreover, FMD reduces the clinical severity of EAE in mice, and these improvements were associated with an increase in the number of regulatory T cells and decreases in the levels of proinflammatory cytokines, TH1 and TH 17 cells, and antigen-presenting cells (Choi et al., 2016). In addition, FMD promotes oligodendrocyte progenitor cell regeneration and myelin regeneration in both MS and EAE models, which supports its inhibitory effect on autoimmunity and myelin regeneration (Choi et al., 2016). Numerous studies have confirmed the effects of CR on systemic immunity and chronic inflammation, although further evidence is needed to validate the effect of CR on neural autoimmunity.

## Caloric Restriction Interventions for Brain Aging and Related Neurodegenerative Diseases

As stated above, the CR paradigm can affect the brain microenvironment at molecular, cellular, and tissue levels. Although most of those mechanistic models were established using rodent models, the beneficial clinical effects of CR have been reported across various disease conditions. In general, CR exerts neuroprotective effects by modulating metabolism and neuroinflammatory environments to preserve the existing neural network, which may help recover synaptic connections to regain neural functions.

### Anti-aging Effect of Caloric Restriction and Neurogenesis Under Normal Homeostasis

Neurogenesis is a process in which progenitor cells develop into intact neurons, and it occurs in embryonic brains and discrete regions of adult brains (Gross, 2000; Lie et al., 2004). In most mammalian species, active neurogenesis occurs throughout life in the SVZ of the lateral ventricle and the subgranular zone of the DG in the hippocampus (Ming and Song, 2005; Qiu G. et al., 2010). FMD can improve cognitive functions by increasing neurogenesis mediated by the protein kinase A/CREB-dependent neuroD1 pathway (Sharma et al., 1999; Brandhorst et al., 2015) and by increasing neuronal survival and differentiation of hippocampus progenitor cells (Roybon et al., 2009). In addition, adult hippocampal neurogenesis is enhanced by CR and is associated with circulating auxin-releasing peptide levels. However, studies have also reported that long-term CR does not delay the age-related decline of hippocampal neurogenesis, but instead increases the survival rate of glial precursors in the hilus (Bondolfi et al., 2004). Further systematic studies are needed to gain a full understanding of the effects of CR on hippocampal neurogenesis.

### Alzheimer’s Disease

During AD pathogenesis, mitochondrial dysregulation is considered a critical factor for maintaining cellular, metabolic, and redox homeostasis, which affects neurite growth and synaptic function (Cheng et al., 2010). CR paradigms have been shown to increase the calcium retention capacity of brain mitochondria (Amigo et al., 2017), which offers a mechanism by which dietary interventions relieve AD-related mitochondrial dysfunction. Moreover, autophagy has been suggested as a mechanism to facilitate the recovery from AD-related symptoms because CR promotes cellular autophagy (Yang and Zhang, 2020). The molecular pathway involved is likely the CR-modulated mTOR pathway (Van Cauwenberghe et al., 2016); its inhibition facilitates cellular autophagy and other biological processes to protect neural tissues from degeneration.

### Parkinson’s Disease

Parkinson’s disease is another common neurodegenerative disease in the aging population. Investigations have reported a correlation between PD risk and dietary habits (Gardener and Caunca, 2018). Using a cell model, CR rescued cells from synucleinopathy (Sampaio-Marques et al., 2018), which is a common feature of PD. In a primate PD model, CR increased NTFs, which attenuated the pathological and behavioral deficits of the disease (Maswood et al., 2004). By suppressing the cellular stress response, CR helps to facilitate NTFs, chaperone proteins, DNA repair, and MB (Mattson, 2012), all of which contribute to the relief of disease symptoms.

### Multiple Sclerosis and Amyotrophic Lateral Sclerosis

Multiple sclerosis is an autoimmune disease that can cause demyelination of the central nervous system, leading to varying degrees of axonal and neuronal damage. IF improves the clinical course and pathology in MS and EAE animal models by relieving demyelination or axonal damage (Cignarella et al., 2018). In MS animal models, IF provides protection by affecting the intestinal microbiota, and similar effects on gut microbiota have been observed in patients with recurrent MS following short-term IF intervention (Cignarella et al., 2018). In a clinical trial, participants who did not follow a low-sugar, low-carb, low-calorie diet were more likely to have progressive MS, accompanied by obesity and weight gain (Fitzgerald et al., 2018). Although the CR diet temporarily improved motor performance in G93A mice, an animal model of amyotrophic lateral sclerosis (ALS), it accelerated the clinical onset of the disease, which suggested that the CR diet may not be a protective strategy for patients with ALS (Hamadeh et al., 2005).

### Huntington’s Disease

As an inherited neurodegenerative disorder, Huntington’s disease (HD) is characterized by the degeneration of neurons in the striatum and cerebral cortex, which results in involuntary movements and abnormalities of psychiatric and cognitive functions (Duan et al., 2003). Both HD patients and transgenic mouse models exhibit reduced levels of BDNF and metabolic disorders, in addition to the mutated human Huntingtin protein. CR increases BDNF levels and the protein concentration of chaperone heat-shock protein-70 in the striatum and cortex and prolongs the life span of HD patients by alleviating neuropathological, movement, and metabolic abnormalities (Duan et al., 2003). Similarly, a ketogenic diet in a mouse model has been shown to interfere with HD, where weight loss was delayed; however, improvements in movement disorders or longevity were not observed (Ruskin et al., 2011). Currently, studies on the detailed molecular mechanism of CR interventions on HD remain limited.

### Traumatic Brain Injury and Stroke

Both TBI and stroke are complex pathological processes that comprise primary and secondary injuries (Lončarević-Vasiljković et al., 2016) and are commonly accompanied by cognitive impairments, which include deficits in attention, processing speed, memory, and executive functions (Rich et al., 2010). CR has been shown to improve behavioral outcomes after ischemic brain injury. IF for 3 months (a 30–40% reduction in food intake) in a middle cerebral artery occlusion reperfusion rat model showed significantly greater improvement in behavioral scores than that in the normal diet group (Yu and Mattson, 1999). The cellular and molecular mechanisms underlying the protection of brain cells against stroke and TBI by CR involve the up-regulated expression of neurotrophic factors (BDNF and FGF2), antioxidant enzymes, and protein chaperones (Arumugam et al., 2010). Decreased leptin and increased ketone levels may also contribute to the neuroprotective effects of CR in stroke models (Manzanero et al., 2014). In addition, CR is currently considered a preventive lifestyle that reduces the severity of TBI outcomes and tissue damage and improves recovery after injury (Roberge et al., 2008; Rich et al., 2010). One study showed that CR for 3 months before TBI eliminates TNFα-dependent caspase-3 activation and secondary neuronal apoptosis, which suggests that DR strongly influences external apoptotic pathways (Loncarevic-Vasiljkovic et al., 2012). However, it was speculated that CR, as an additional external pressure, further aggravates the energy crisis caused by TBI and stroke (Lončarević-Vasiljković et al., 2016).

### Retinal Aging

In retinal aging, CR protects the structural and functional integrity of retinal ganglion cells by facilitating cellular autophagy, which improves the progression of glaucoma that occurs commonly in aging people (Adornetto et al., 2020). In regard to age-related changes in retinal tissues, CR has been shown to reduce certain metabolites, such as glutathione and ascorbic acid levels (Li et al., 2003), exerting a protective function against retina degeneration. Such metabolic regulatory effects may be related to transcriptional factors such as nuclear factor-erythroid factor 2-related factor 2 (Nrf2; Izuta et al., 2018). In the future, additional studies to investigate the value of CR in counteracting retinal aging are warranted.

We have summarized the major findings regarding the application of CR in alleviating neurodegenerative diseases (Table 2). In practice, dietary plans may be combined with anti-aging drugs to synergistically counteract brain degeneration. For example, resveratrol has been found to function as a mimetic compound that partially substitutes the effects of CR (Barger, 2013). In addition, a similar observation was made for the SIRT1 activator drug, SRT1720 (Suzuki et al., 2012), which is known to play a role in increasing longevity induced by CR. Further knowledge can be gained from neuroendocrine studies, such as the role of ghrelin in mediating CR-induced neuroprotective effects (Bayliss et al., 2016). Taken together, further investigations on the mechanisms underlying the neuroprotective effects of CR may reveal potentially additive effects of combing both drugs and dietary plans, which will enable the development of more effective interventions.

**TABLE 2:** Recent studies of dietary interventions for different neurodegenerative diseases.

**Disease and model**	**Diet scheme**	**Major findings**	**References**
**Alzheimer’s disease (AD)**			
AD mouse model	Intermittent food deprivation	Improved cognitive and mental functions and protected neurons.	Liu Y. et al., 2019
Aging people (58–98 years)	MIND diet	Decreased long-term AD risk.	Morris et al., 2015
Individuals with normal cognitive function (30–60 years)	Mediterranean diet	Protection against brain aging and AD.	Berti et al., 2018
Transgenic AD mouse model (APP/PS1)	Caloric restriction (CR)	CR decreased approximately 1/3 of total Aβ volume in the cortex.	Mouton et al., 2009
AD mouse model	Dietary restriction (DR)	Relieved Aβ load and memory deficits.	Gregosa et al., 2019
Female Tg2576 Aβ AD mice	Calorie restriction (CR)	Long-term CR relieved pathology, probably dependent on γ-secretase induced amyloid precursor protein (APP) inhibition.	Schafer et al., 2015
**Parkinson’s disease (PD)**			
Yeast cell expressing human α-synuclein (SNCA)	Caloric restriction (CR)	Regulation of ubiquitinase and cellular autophagy.	Sampaio-Marques et al., 2018
SNCA transgenic mouse	Alternate day fasting diet	DR may relieve autonomic nervous system dysfunction.	Griffioen et al., 2013
Intra-striatal injection of 6-OHDA (Rat)	Alternate day fasting diet	No significant effect on neuroprotection or behavior.	Armentero et al., 2008
**Amyotrophic lateral sclerosis (ALS)**			
ALS model mice (G93A)	Caloric restriction	Temporally improved motor dysfunctions but may accelerate disease progression long-term.	Hamadeh et al., 2005
**Multiple sclerosis (MS)**			
MS mouse model	Intermittent fasting (IF)	Effective immune-modulatory effects, partially induced by gut microbes.	Cignarella et al., 2018
MS patients	A total of 19 different diets	People with no specific diet plan may have a higher risk of MS.	Fitzgerald et al., 2018
**Huntington’s disease (HD)**			
HD mutant mice	Dietary restriction	DR enhanced BDNF and HSP70 levels in the striatum and cortex, which improved HD symptoms.	Duan et al., 2003

## Conclusion and Future Perspectives

The extracellular microenvironment is critical for maintaining normal physiological functions of cells because of its role in the homeostatic regulation of various components. Various factors, such as inflammation, metabolic waste, and the BBB, can disrupt normal brain microenvironments. Thus, the maintenance of the extracellular environment is vital for brain health. Current studies have suggested that lifestyle interventions, such as regular exercise training, a healthy diet, and sufficient sleep, protect the brain by improving the microenvironment balance under pathological conditions.

Caloric restriction effectively protects the brain microenvironment via multiple mechanisms at molecular, cellular, and tissue levels. Major benefits obtained by CR are based on recent findings in aging and neuropathological models. However, there is currently no consensus on a unified protocol for CR because the duration or starting age of CR has not been clarified in animals. Currently, IF and CR are the two predominant approaches. For the CR scheme, several precautions need to be specified, which include the avoidance of artificial sweeteners and the supplementation of probiotics. Specifically, the ketogenic diet has been shown to significantly affect gut microbes, whereby its composition is prominently altered to induce metabolic disorders, leading to brain dysfunctions (Paoli et al., 2019). Thus, further investigations on the gut microbial diversity under the CR paradigm are needed to develop guidelines for monitoring and treating the gut–brain axis.

Reports have shown that the initiation age and duration of CR are critical factors that influence overall efficiency. Specifically, CR started at middle-age has the most potent neuroprotective effect (Todorovic et al., 2018). Current studies on the neuroprotective effects of CR have various weaknesses, which include the lack of a precise description of the dosage curve (i.e., the relationship between CR duration and overall efficiency of neuroprotection), the lack of systematic observations of the additive effect of CR and drugs in counteracting neurodegeneration, and the absence of a neural circuit-specific effect of dietary interventions. These factors limit the large-scale promotion of CR in aging and high-risk populations with neurodegenerative diseases. Therefore, future explorations are required to understand the neuroprotective mechanisms underlying CR to develop alternative pharmaceutical or non-drug interventions for brain aging and neurodegeneration.

## Author Contributions

ND prepared materials for the writing. ND, XL, and LZ wrote the manuscript. LZ and ZC revised the manuscript. All authors contributed to the article and approved the submitted version.

## Conflict of Interest

The authors declare that the research was conducted in the absence of any commercial or financial relationships that could be construed as a potential conflict of interest.

## Publisher’s Note

All claims expressed in this article are solely those of the authors and do not necessarily represent those of their affiliated organizations, or those of the publisher, the editors and the reviewers. Any product that may be evaluated in this article, or claim that may be made by its manufacturer, is not guaranteed or endorsed by the publisher.
